# Realization of a thermal cloak–concentrator using a metamaterial transformer

**DOI:** 10.1038/s41598-018-20753-y

**Published:** 2018-02-06

**Authors:** Ding-Peng Liu, Po-Jung Chen, Hsin-Haou Huang

**Affiliations:** Department of Engineering Science and Ocean Engineering, National Taiwan University, Taipei, 106 Taiwan

## Abstract

By combining rotating squares with auxetic properties, we developed a metamaterial transformer capable of realizing metamaterials with tunable functionalities. We investigated the use of a metamaterial transformer-based thermal cloak–concentrator that can change from a cloak to a concentrator when the device configuration is transformed. We established that the proposed dual-functional metamaterial can either thermally protect a region (cloak) or focus heat flux in a small region (concentrator). The dual functionality was verified by finite element simulations and validated by experiments with a specimen composed of copper, epoxy, and rotating squares. This work provides an effective and efficient method for controlling the gradient of heat, in addition to providing a reference for other thermal metamaterials to possess such controllable functionalities by adapting the concept of a metamaterial transformer.

## Introduction

The concept of controlling energy can be traced back to the pioneering proposals by Pendry^1^ and Leonhardt^2^, whose approaches to cloaking provided means of manipulating electromagnetic waves. Following their research, efforts have been devoted to modeling and designing coordinate transformation-based metamaterials, also known as thermal metamaterials^3–5^, in the field of thermodynamics. Owing to the unconventional nature of thermal metamaterials, various applications such as the thermal cloak^6–14^, thermal concentrator^15–17^, thermal inverter^18–20^ and thermal illusion^21–24^ have been proposed.

Recently, thermoelectric components^25^ have offered a method for actively controlling heat flux. Other alternatives such as the shape memory alloy^26^ have also been demonstrated, theoretically and experimentally, for use as a thermal diode^26^, a thermal cloak–concentrator (TCC)^27^, and a temperature-trapping device^28^. Analogous to the thermal cloak and concentrator, a practical idea for adapting the concept of manipulating the heat flow in electronic components has been applied^29,30^. In this method, the temperature within a shield area can be reduced and the concentrator can be employed to collect the low-grade waste heat of electronic components. Consequently, thermal metamaterials have been considered an important subject for future technology and applications. However, fabrication of such materials is extremely challenging owing to the continuous change in their thermal properties. The notion of discretizing thermal metamaterials into unit-cell thermal shifters, representing heat flux lines in local spots, was therefore recently proposed^31^. The method not only simplifies the manufacture of thermal metamaterials but also maintains their functionalities, including the cloak, concentrator, diffuser, and rotator.

Recent research on thermal metamaterials has thus focused on the controllability of functionalities. In this study, we propose a new class of thermal metamaterials, namely a metamaterial transformer (MMT), by combining unit-thermal shifters and rotating squares^32,33^, a form of auxetic metamaterials^34^. Rotating squares become thicker as the rotation angle increases to 45°. They gradually close and shrink until the rotation angle is 90°. The rotating squares remain in the same configuration, whereas the unit cells of the rotating squares rotate 90° relative to each other. The proposed thermal metamaterials can possess tunable functionalities by transforming the configuration of the device. The designed MMT acts as a type of TCC that initially works as a cloak and then as a concentrator after the rotation of all squares. In contrast to previous approaches that are based on shape memory alloys, our MMT-based TCC can realize dual functions that can be freely controlled without out-of-plane deformation. We then developed a theoretical model of the MMT-based TCC for predicting the property of the TCC after the rotating squares have rotated 90°. In the following section, we demonstrate the cloaking and concentrating effect of the proposed device in both simulations and experiments.

## Results

### Numerical and experimental setup

The commercial finite element software COMSOL Multiphysics 5.3 was used for numerical simulations in this study. We performed simulations with a two-dimensional heat conduction module under a steady-state condition. The MMT-based TCC was analyzed in the following three cases. First, a theoretical model was constructed to simulate the functionality of an ideal TCC and was then rotated 90°. Second, an effective model composed of unit cell thermal shifters with a contact interface was constructed for verification. Finally, a revised effective model of the MMT-based TCC was constructed to take radiation heat loss into consideration.

**Figure 1:**
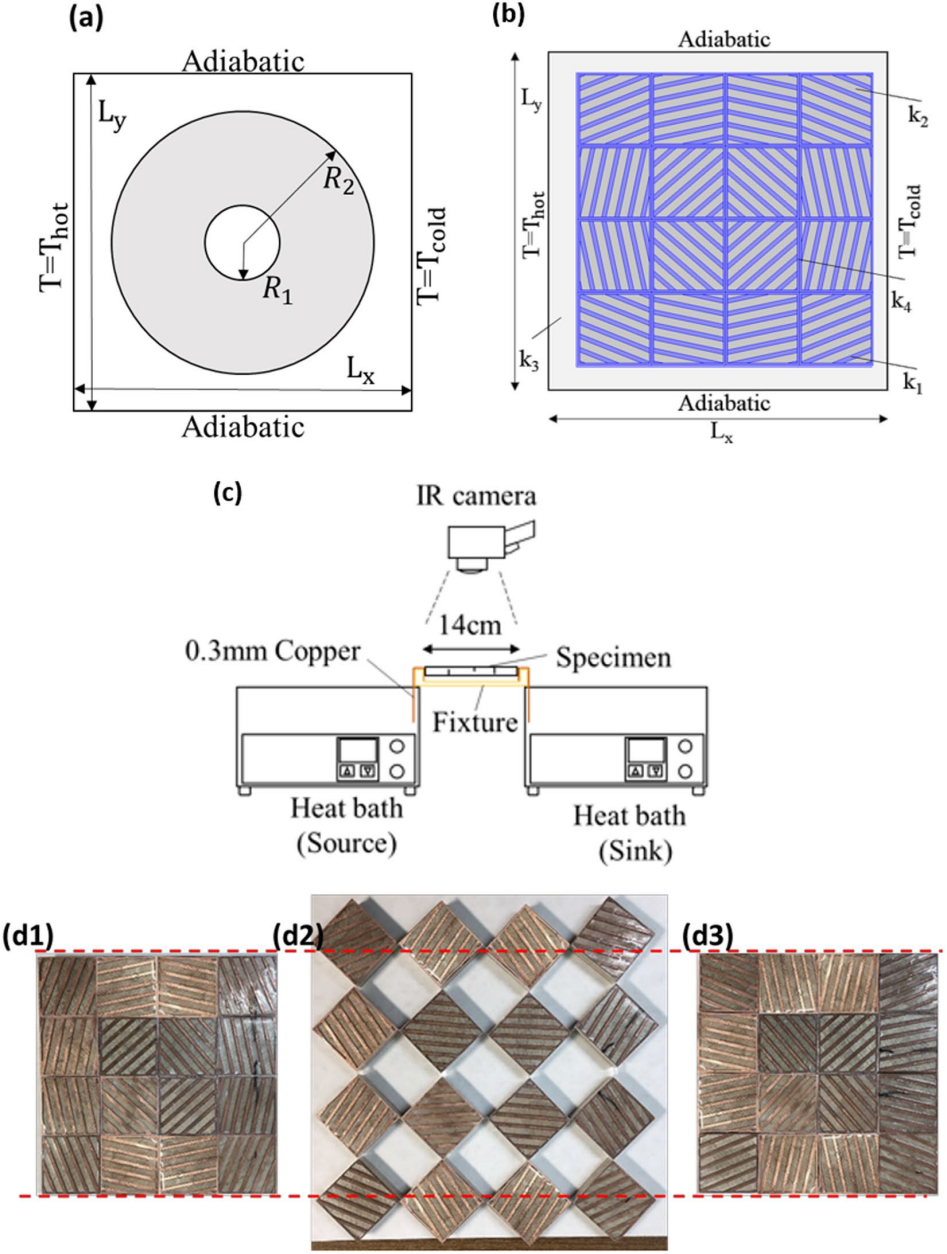
Schematic of numerical simulation and experimental setup. (**a**) Boundary conditions and geometrical dimensions of the theoretical model. (**b**) Boundary conditions and geometrical dimensions of the effective models. (**c**) Side view diagram of the experimental apparatus, illustrating the method for device temperature measurement through IR thermography. (**d1**), (**d2**), and (**d3**) MMT-based TCC with rotation angles of 0°, 45° and 90°, respectively.

For the theoretical model, we set the top and bottom surfaces as the insulators, whereas the left and right boundaries were prescribed temperatures in accordance with the experimental measurements. The model comprised a background material with thermal conductivity *k* = 1*W*/(*mK*) in both the interior and exterior of the circle, and the circular material was given theoretical anisotropic thermal conductivity (see Methods) with ${R}_{2}=70\sqrt{2}\,\,{\rm{mm}}$, ${R}_{1}=\frac{35}{2}\sqrt{2}\,\,{\rm{mm}}$ and *L*_*x*_ = *L*_*y*_ = 210 mm, as shown in Fig. 1(a).

The effective TCC model was constructed based on Supplementary Tables S1 and S2. The thermal conductivity of copper, epoxy and background materials were defined as *k*_1_, *k*_2_, and *k*_3_, respectively. The boundary conditions were set identical to those in the theoretical model, and the configuration of the proposed TCC is shown in Fig. 1(b), with *k*_1_ = 400*W*/(*mK*), *k*_2_ = 0.3*W*/(*mK*), *k*_3_ = 90*W*/(*mK*), *L*_*x*_ = *L*_*y*_ = 210 mm and *l*_*x*_ = *l*_*y*_ = 140 mm. Moreover, we further considered mimicking the experimental setup by adding a thin layer of thermal compound in between adjacent unit cells, and set the thin-layer (0.1 mm) thermal compound with a low thermal conductivity *k*_4_. The choice of *k*_4_ is described in Supplementary Section 1 and discussed later.

The experimental setup consisted of a thermal infrared (IR) camera (Fluke Ti-450), heat baths (Aron WB-500D), a fixing device, and a specimen, as shown in Fig. 1(c). The heat bath on the left side served as the source applied to the specimen, whereas the heat bath on the right side, filled with an ice–water mixture, was used as the heat sink. The specimen was assembled into 4 × 4 unit-cell thermal shifters composed of copper and epoxy, with 4 × 4 rotating squares serving as the base connected with joints, as shown in Fig. 1(d1),(d2), and (d3). The design of joints connecting the rotating squares in the present study is illustrated in Supplementary Figure S2. In addition, the assembled specimen was connected to the thin copper plate with 0.3 mm thickness at each end. We polished the interfaces of the unit-cell thermal shifters and added the thermal compound (Cooler Master RG-ICF-CWR2-GP) (Thermal conductivity *k* = 1*W*/(*mK*), from the product sheet provided by the manufacturer) in between adjacent unit cells to reduce the thermal resistance. As illustrated in Fig. 1(c), the temperature profile on the topside of the specimen was obtained through a calibrated IR camera, with a thin coating of high-emissivity (ε = 0.94) black acrylic paint, ART-ANDREA-S-72075801, applied on the topside for accurate thermal imaging.

### Numerical simulations of MMT-based TCC

**Figure 2:**
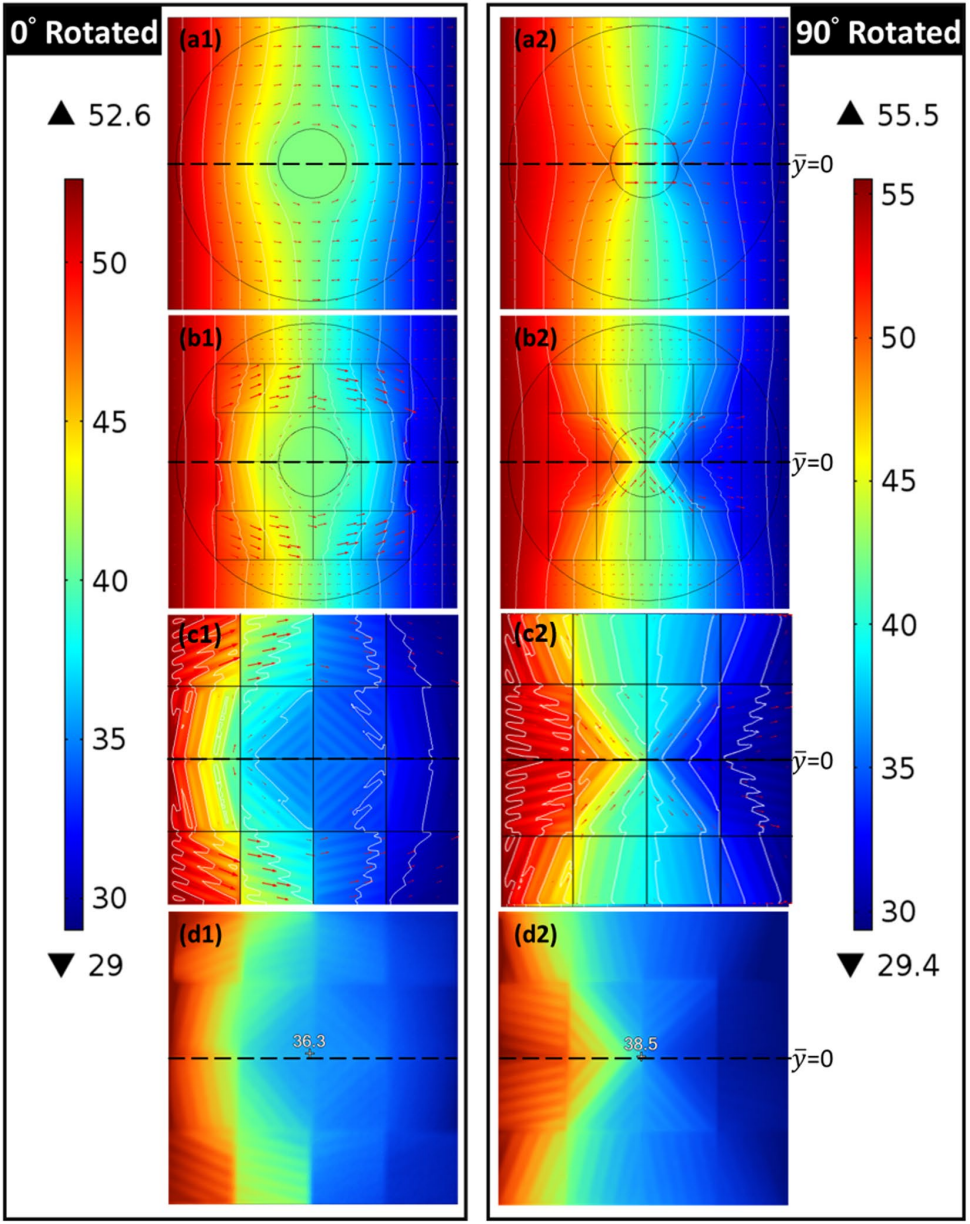
(**a1**), (**b1**), (**c1**), and (**d1**), and (**a2**), (**b2**), (**c2**), and (**d2**) depict simulated temperature profiles and isothermal lines of the theoretical, effective, and revised models and the measured temperature profiles of the TCC, respectively, with red arrows indicating the heat flux through the model in 0° rotation (90° rotation). The applied fixed temperature is shown on the color bar, and the annular rings represent the geometry of the theoretical model and the square lines represent the effective model (The Celsius scale is used for the unit of temperature).

The results of the theoretical model are summarized in Fig. 2(a). Figure 2(a1,a2) illustrate the simulated temperature profile and isothermal lines of the theoretical model, respectively, with the red arrows indicating the directions of the heat flux. The simulation revealed the properties of the proposed TCC, where no external distortion existed and no internal gradient was observed (no heat flux through the inner region). After the specimen was rotated 90°, no external distortion existed, but a much greater internal gradient was observed (with greater heat flux through the inner region), as shown in Fig. 2(a1,a2).

Figure 2(b1,b2) present the temperature profile and isothermal line of the TCC, respectively. An annular ring of the theoretical model was plotted to clarify the difference between the theoretical and effective models. As illustrated in Fig. 2(b1), the temperature inside the annular ring was almost constant, with the isothermal lines outside the annular ring exhibiting minimal disturbance, similar to the properties displayed by a thermal cloak. As shown in Fig. 2(b2), the model rotated 90°, demonstrating the ability to guide the heat flux into the inner region of the annular ring, thus causing more temperature variations inside the annular ring and a slight disturbance outside the ring.

The preceding results show that the proposed MMT-based TCC can control the gradient within a particular region. However, it is reasonable to question whether such an ability can still exist when the radiation heat loss as well as the thermal resistance in the contact interfaces are considered. To explore this question numerically, in the final case we applied a surface-to-ambient radiation boundary condition in COMSOL; that is ${\rm{Q}}={\varepsilon }_{u}\sigma ({T}_{amb}^{4}-{T}^{4})$, where Q is the thermal energy leaving the surface, ε_*u*_ = 0.94 is the emissivity of the surface, σ is the Stefan–Boltzmann constant, and *T*_*amb*_ = 28.9 °C is the ambient temperature. To compare the experimental results, the revised effective model and the experimental specimen were constructed as similar as possible. For the revised model, we simultaneously simulated the thermal radiation with the thermal resistance due to the contact interface (see Supplementary Section 1) and concluded that the temperature profile along $\bar{y}=0$ when setting k = 0.5 W/(mK) is closest to that of the experimental data. Figure 2(c1,c2) show the simulated temperature profile and isothermal lines of the revised effective model, respectively. The isothermal lines detoured around the inner region in the model rotated 0°, and were compressed into the inner region in the model rotated 90°. Clearly, when the radiation heat loss was considered, the prominent control of the gradient within the inner region varied when rotated either 0° or 90°.

### Experimental validation of MMT-based TCC

The steady-state temperature profile of the specimen was then measured using the IR camera, and the results of which are shown in Fig. 2(d1,d2). The temperature of the inner region was almost constant, as shown in Fig. 2(d1), but it changed drastically, as shown in Fig. 2(d2). However, owing to heat loss to the surrounding area, the overall temperature was lower near the right side of the specimen, which was determined to be consistent with the revised model in Fig. 2. Comparisons of the theoretical, effective, revised model, and experimental results of the TCC are discussed further.

## Discussion

**Figure 3:**
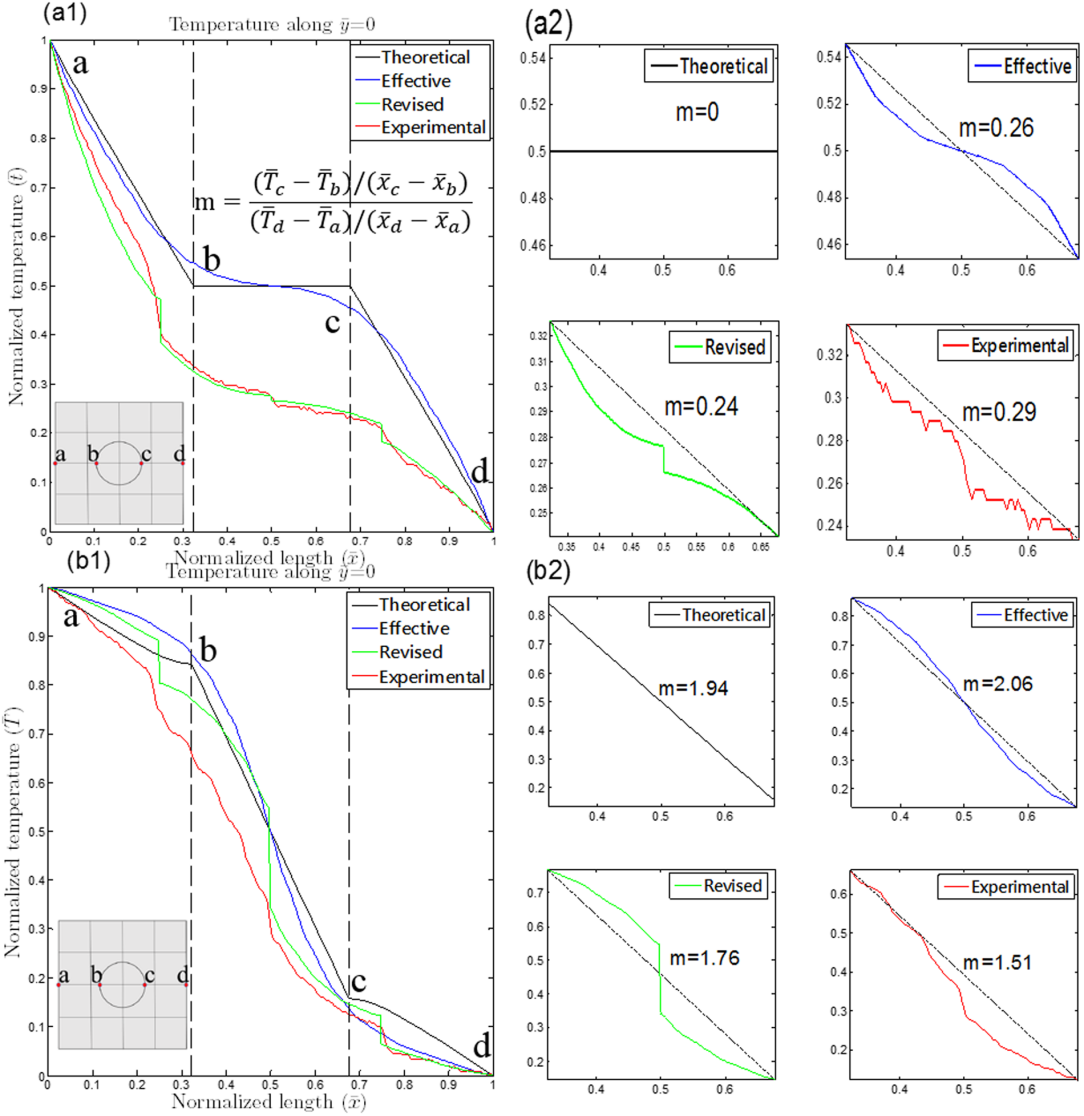
(**a1**) and (**b1**) present temperature along $\bar{y}=0$ of simulated and experimental results in both 0° and 90° rotated TCCs, where broken lines indicate the inner region of the TCC. The gradient within line segment $\overline{bc}$ over the applied gradient is plotted in (**a2**) and (**b2**) for clearer observation.

First, the theoretical and effective models of the TCC, along with the revised model and the experimental results, are discussed. From Fig. 2, the temperature along $\bar{y}$ = 0 in all model types and experimental results can be plotted, as shown in Fig. 3, in which the broken lines indicate the inner region of the TCC. We noted that the effective model was consistent with the theoretical model, as shown in both Fig. 3(a1,b1). This means that the proposed MMT-based TCC is equivalent to the theoretical model deduced from transformation thermodynamics. However, the overall temperature in the experimental results was lower than that observed in the effective model, as shown in both Fig. 3(a1,b1). This implies a loss of heat into the ambient. Furthermore, the thermal interfacial resistance present in the contact interface of the thermal shifters also caused a considerable amount of heat loss to the surroundings.

For further comparison, the ability to change the temperature gradient inside the proposed TCC is presented in Fig. 3(a2,b2), where ${\rm{m}}=\frac{({\bar{T}}_{c}-{\bar{T}}_{b})}{({\bar{x}}_{c}-{\bar{x}}_{b})}/\frac{({\bar{T}}_{d}-{\bar{T}}_{a})}{({\bar{x}}_{d}-{\bar{x}}_{a})}$, is the gradient of the temperature within line segment $\overline{{\rm{bc}}}$ over the applied gradient. We noted that the performance of cloaking improved when the device had a small value of m; moreover, a large value of m indicated better performance for the concentrator. As illustrated in Fig. 3(a2), when the device was rotated 0°, the m value was 0 for the theoretical model, 0.26 for the effective model, and 0.24 for the revised model, whereas the measured result was 0.29. Because the overall temperature was lower in the revised model and experiment, the m value of the revised model was lower than that of the effective model. However, due to thermal interfacial resistance of the thermal compound and rough contact interfaces, a sudden drop in temperature occurred at $\bar{x}=0.5$, causing a slight increase in m. Furthermore, as shown in Fig. 3(b2), when the device was rotated 90°, the m value was 1.94 for the theoretical model, 2.06 for the effective model, and 1.76 for the revised model, whereas the measured result was 1.51. Notably, the gradient of the effective model was even greater than that of the theoretical model, a result caused by the fact that the inner region of the theoretical model was composed of background material which had no ability to concentrate the heat. Moreover, the thermal resistance of unit-cell thermal shifters resulted in a lower m value, compared with the revised model.

**Figure 4:**
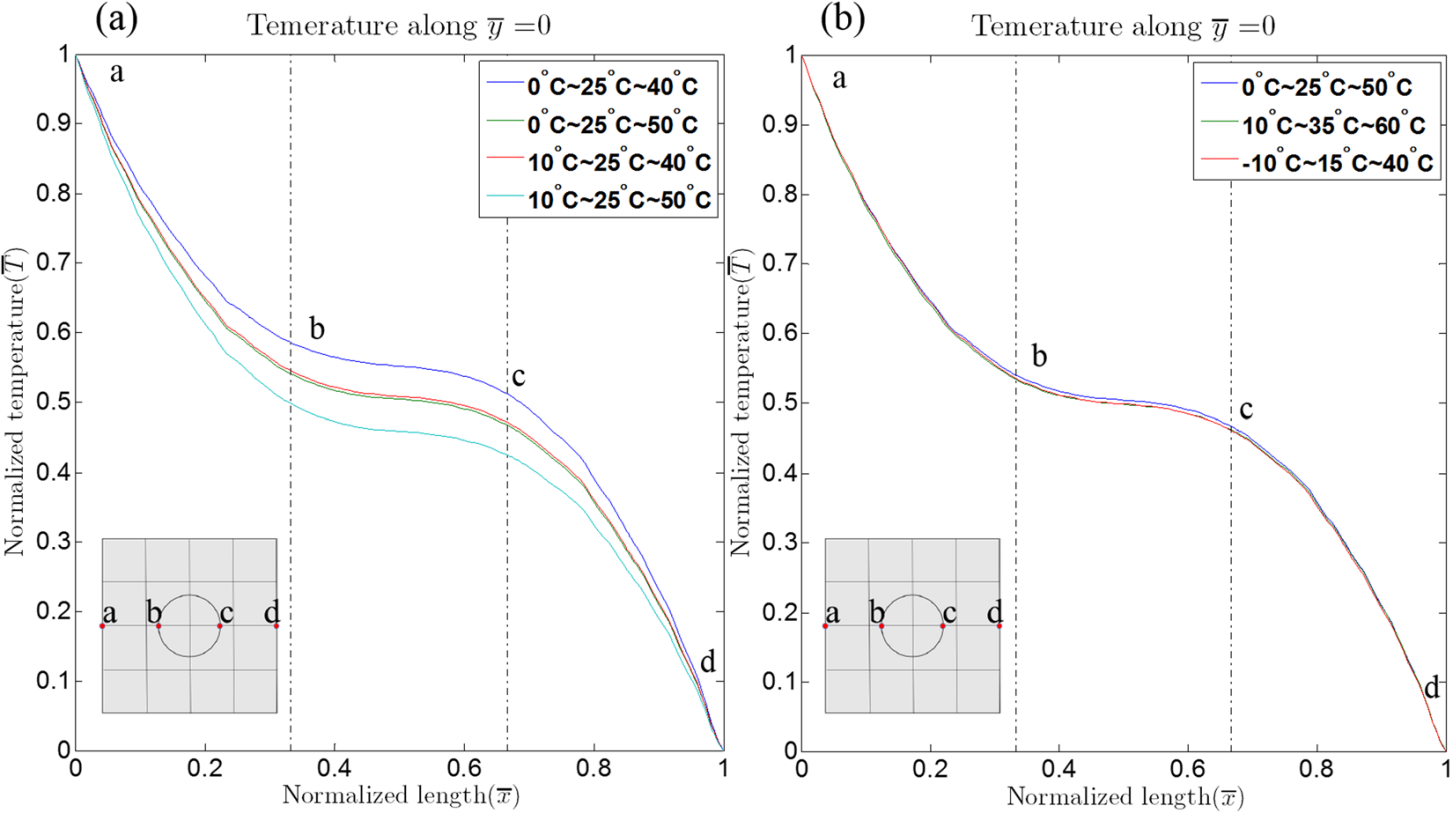
Simulated temperature along $\bar{{\rm{y}}}=0$ in the 0° rotated TCC for (**a**) various modulation of temperature ranges with fixed surrounding temperature, and (**b**) fixed temperature range with central temperature equal to the surrounding temperature. The temperatures shown in the legend, for instance, “X°C~Y°C~Z°C” represents the heat sink (X), the surrounding (Y), and the heat source (Z) temperatures.

The effect of the modulation of the temperature range and the surrounding temperature due to radiation was numerically examined. Specifically, the 0° rotated TCC was investigated. First, the surrounding temperature in the model was fixed at 25 °C, and the modulation of the temperature range was varied. The simulation results, as shown in Fig. 4(a), reveals that the overall normalized temperature in the cloaking zone decreases when the surrounding temperature is close to the lower-bound temperature (See the case: 10 °C ~25 °C~50 °C), and vice versa. The normalized temperature profile along $\,\bar{y}$ = 0, however, behaves similarly as the surrounding temperature and the modulation of the temperature range have the same deviation. (Fig. 4(b)).

**Figure 5:**
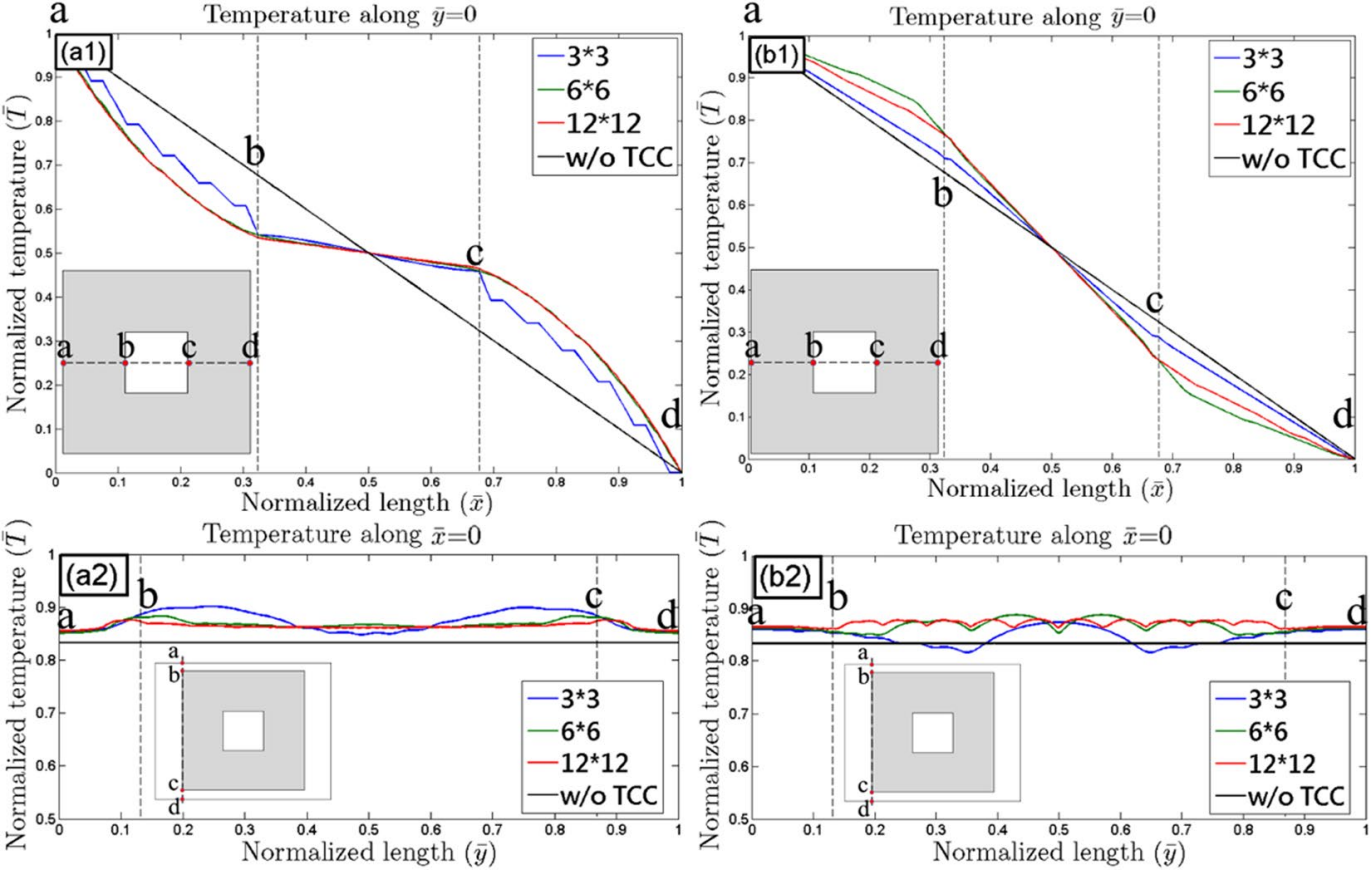
Temperature along (**a1**) (**b1**) $\bar{y}=0$, (**a2**) (**b2**)$\,\bar{x}=0$, when rotated 0° (90°).

**Figure 6:**
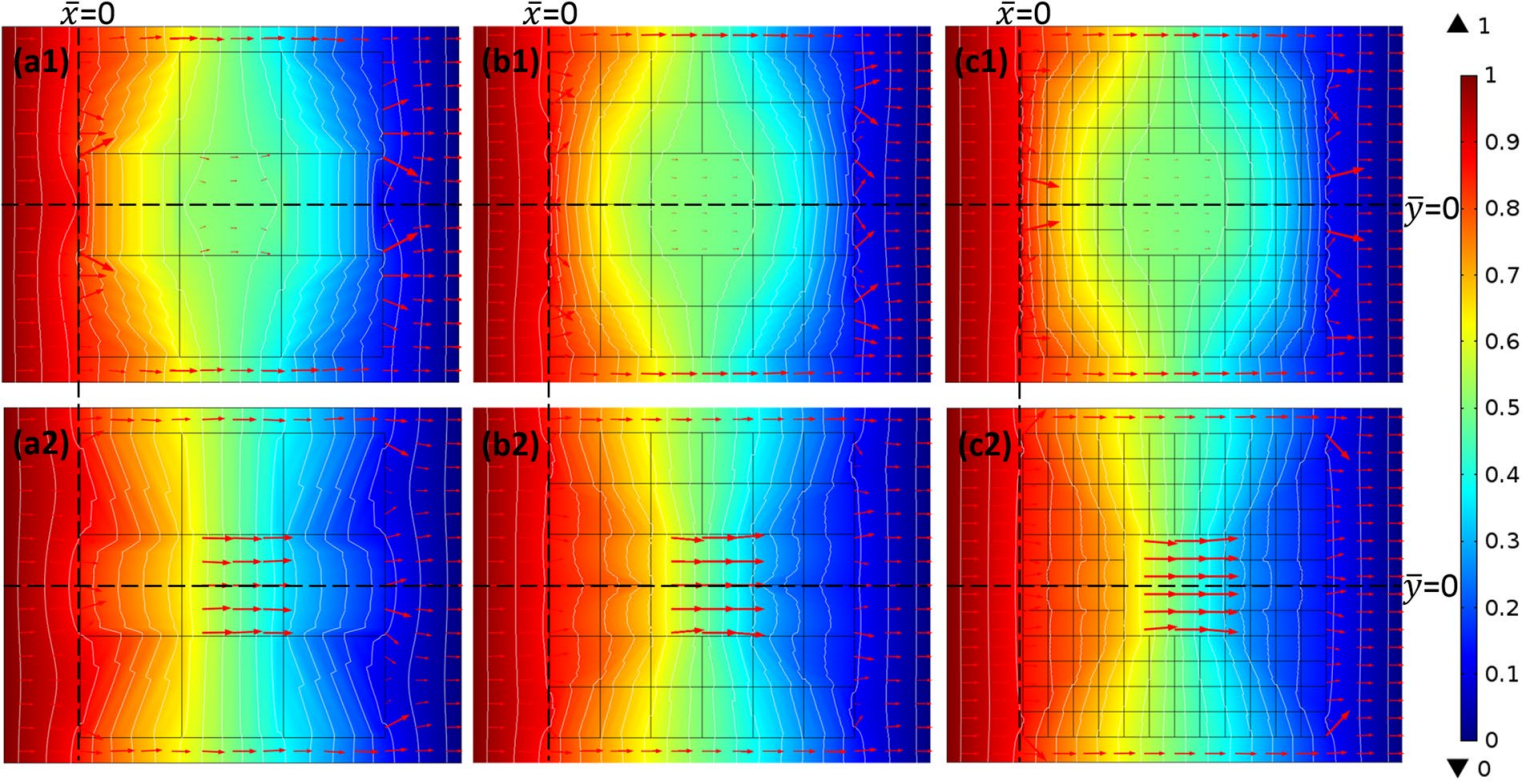
Temperature profile and isothermal lines of different numbers of thermal shifters: 3 × 3 rotated by (**a1**) 0° and (**a2**) 90°; 6 × 6 rotated by (**b1**) 0° and (**b2**) 90°; 12 × 12 rotated by (**c1**) 0° and (**c2**) 90°.

For application considerations, the unit cells of the inner region of the effective model in the simulation were removed, and the number of unit-cell thermal shifters is discussed in this section. The temperature along $\bar{y}$ = 0 is shown in Fig. 5, in which the broken line indicates the inner region of the TCC. In the device rotated 0°, we noted that when the number of unit cells increased, the gradient within the inner region decreased, as shown in Fig. 5(a1); by contrast, in the device rotated 90°, the gradient increased as the number of unit cells increased, as shown in Fig. 5(b2). For ease of comprehension, Table 1 shows the gradient within line segment $\overline{bc}$ over the applied gradient, m. We observed that the ability to change the gradient internally was below the expected level in the 3 × 3 unit cell-based TCC, because the number of unit cells in the model was insufficient for the anisotropic thermal conductivity to be deduced from transformation thermodynamics. However, once the number of unit cells was sufficient, for example 6 × 6, an ability to change the gradient was then demonstrated. Moreover, the isothermal distortion of the exterior of the device can be observed in Fig. 5(a2,b2) and Fig. 6(a1,a2,b1,b2,c1,c2). The isothermal distortion was observed to decrease when the number of unit-cell thermal shifters increased in both 0° and 90° rotated TCCs.

**Table 1:** Gradient within line segment $\overline{bc}$ over the applied gradient.

	3*3	6*6	12*12
0° rotated	0.24	0.22	0.19
90° rotated	1.24	1.53	1.54

## Conclusion

By combining rotating squares with auxetic property, we proposed the use of thermal metamaterials with tunable functionalities. An MMT-based TCC, which can change from a cloak to a concentrator when the device configuration is transformed, was then investigated. The MMT-based TCC can thermally protect a region and enable a concentrator to focus heat flux in a small region, and this was verified in both simulations and experiments.

In summary, the proposed MMT-based TCC could control the gradient within the inner region in both the theoretical and the effective models. However, this ability was slightly suppressed when the radiation heat loss considered, which is reasonable given that the transformation thermodynamics is based on the theory of heat conduction. However, the contact interface of the thermal shifters should be sufficiently smooth to minimize thermal interfacial resistance. Additionally, for application considerations, we removed the unit cells of the region within the device and found that the greater the number of unit cells, the better the ability to control the gradient within the device (and vice versa). Furthermore, when the number of thermal shifters was sufficient, we observed only a small amount of isothermal distortion outside the device. With such functionality to control the gradient internally, the proposed TCC can be used for thermal protection of a heat-sensitive device or as a thermal concentration application within a given region.

## Methods

**Figure 7:**
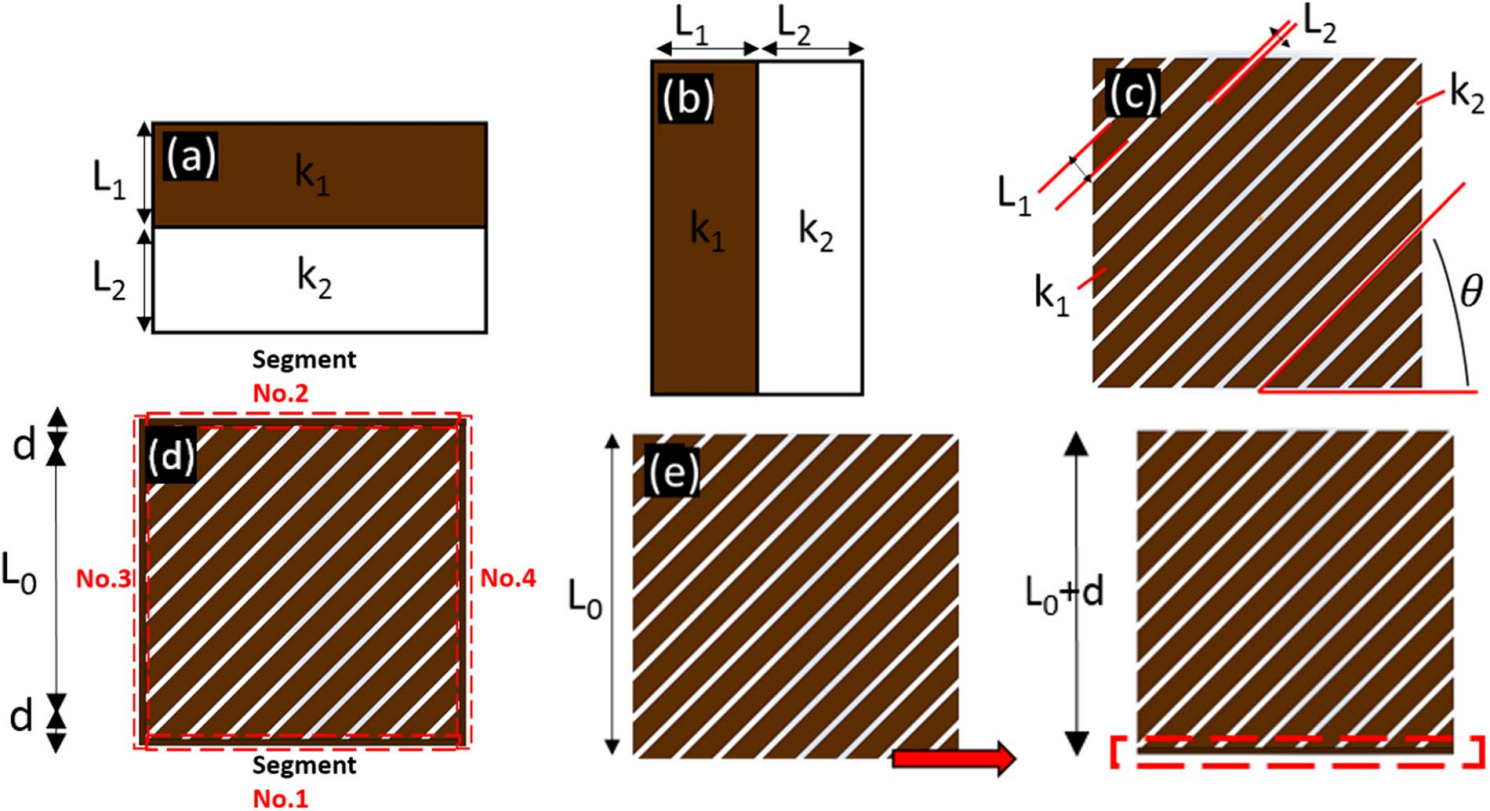
Stacking materials with different thermal conductivity levels in (**a**) parallel and (**b**) series. (**c**) Specific parameters of a unit-cell thermal shifter rotated by *θ*. (**d**) Unit-cell thermal shifter with contact interfaces divided into four segments. (**e**) Demonstration of the derivation of the effective thermal conductivity of thermal shifters with Segment No.1 from a thermal shifter without contact interface.

In this study, we adapted effective medium theory to construct the unit-cell thermal shifters. The theoretical and effective models of a unit-cell thermal shifter are illustrated in Supplementary Figure S4. The effective thermal conductivity of the composite was fabricated by alternately stacking two sheets of thermal conductivities and can be obtained by Bandaru^35^ as follows:

1 $${k}_{EMT}=[\begin{array}{cc}{{\rm{k}}}_{{\rm{p}}} & 0\\ 0 & {{\rm{k}}}_{{\rm{s}}}\end{array}]$$

where ${k}_{p}=\frac{{k}_{1}{l}_{1}+{k}_{2}{l}_{2}}{{l}_{1}+{l}_{2}}$ is the parallel thermal conductivity, as shown inFig. 7(a), and ${k}_{s}=\frac{{k}_{1}{k}_{2}({l}_{1}+{l}_{2})}{{l}_{1}{k}_{2}+{l}_{2}{k}_{1}}$ is the thermal conductivity in series, as shown in Fig. 7(b). The effective thermal conductivity of the composite rotated in the x-y plane by θ can be given as follows:

2 $${k}_{EMT}^{\text{'}}=\frac{J{k}_{EMT}\cdot Trans(J)}{{\rm{\det }}(J)}$$

where J is the Jacobian for the rotation, and Trans(J) denotes the transpose of J. Using the equation (2), we can obtain the effective thermal conductivity of the thermal shifters composed of alternately stacking two sheets of thermal conductivities rotated by θ, as shown in Fig. 7(c).

However, for the fabrication of thermal metamaterials using the assembly concept, the contact interface between thermal shifters will change the effective thermal conductivity of the thermal shifters. Therefore, the effective thermal conductivity of the thermal shifters with contact interface must be derived. Using the equation (2), we can obtain the following:

3 $${k^{\prime} }_{EMT}=\frac{J{k}_{EMT}Trans(J)}{{\rm{\det }}(J)}=[\begin{array}{cc}{{\rm{k}}^{\prime} }_{{\rm{p}}} & {{\rm{k}}}_{{\rm{xy}}}\\ {{\rm{k}}}_{{\rm{xy}}} & {{\rm{k}}^{\prime} }_{{\rm{s}}}\end{array}]$$

where ${{\rm{k}}^{\prime} }_{{\rm{p}}}={k}_{p}-{k}_{p}{\sin }^{2}\theta +{k}_{s}{\sin }^{2}\theta$, k_xy_ = −(sin2*θ*(*k*_*p*_−*k*_*s*_))/2, and ${{\rm{k}}^{\prime} }_{{\rm{s}}}={k}_{s}+{k}_{p}{\sin }^{2}\theta -{k}_{s}{\sin }^{2}\theta$. We then use the same technique in deriving the equation (1). First, we consider the thermal shifter surrounded by the contact interface as a unit cell, and then divide the contact interface into four segments, as shown in Fig. 7(d). Hence, we can obtain the effective thermal conductivity of thermal shifters with Segment No.1, using the equation (1):

4 $${k}_{EMT1}^{\text{'}}=[\begin{array}{cc}{k}_{p1} & {k}_{xy}\\ {k}_{xy} & {k}_{s1}\end{array}]$$

where ${k}_{p1}=\frac{{k}_{p}^{\prime} {L}_{0}+{k}_{0}d}{{L}_{0}+d};\,{k}_{{\rm{s}}1}=\frac{({L}_{0}+d){k}_{s}^{\prime} {k}_{0}}{{L}_{0}{k}_{0}+d{k}_{s}^{\prime} }$, as shown in Fig. 7(e). Using the same technique, we can obtain the effective thermal conductivity of a thermal shifter with contact interface, ${k}_{EMT4}^{\text{'}}:$

5 $${k}_{EMT4}^{\text{'}}=[\begin{array}{cc}{k}_{p4} & {k}_{xy}\\ {k}_{xy} & {k}_{s4}\end{array}]$$

where ${k}_{p4}=\frac{{k}_{s3}({L}_{0}+d)+{k}_{0}d}{{L}_{0}+2d}$, ${k}_{{\rm{s}}4}=\frac{({L}_{0}+2d){k}_{p3}{k}_{0}}{({L}_{0}+d){k}_{0}+d{k}_{p3}}$, ${k}_{p3}=\frac{{k}_{s2}{L}_{0}+{k}_{0}d}{{L}_{0}+d},{k}_{s3}=\frac{({L}_{0}+d){k}_{p2}{k}_{0}}{{L}_{0}{k}_{0}+d{k}_{p2}}$, ${k}_{p2}=\frac{{k}_{p1}({L}_{0}+d)+{k}_{0}d}{{L}_{0}+2d}$, and ${k}_{s2}=$ $\frac{({L}_{0}+2d){k}_{s1}{k}_{0}}{({L}_{0}+d){k}_{0}+d{k}_{s1}}$. The temperature profiles obtained from (5) are shown in Supplementary Figures S5 and S6.

Once the thermal conductivity of a unit-cell thermal shifter with contact interface is obtained, the discretized thermal cloak can be designed. According to Gueeanu^4^, the anisotropic thermal conductivity of a thermal cloak is given as follow:

6 $$k\text{'}=[\begin{array}{cc}\frac{{\boldsymbol{r}}^{\prime} -{{\boldsymbol{R}}}_{1}}{{\boldsymbol{r}}^{\prime} } & 0\\ 0 & \frac{{\boldsymbol{r}}^{\prime} }{{\boldsymbol{r}}^{\prime} -{{\boldsymbol{R}}}_{1}}\end{array}]k$$

where r′=*R*_1_+*r* (*R*_2_ − *R*_1_)/*R*_2_, and *R*_1_ and *R*_2_ are the inner radius and outer radius of the cloak, respectively. First, we let *R*_2_/*R*_1_ = 4 and ${R}_{2}=70\sqrt{2}\,$mm; thus, the length of the thermal shifter is 35 mm. Substituting these values into the equation (6), we obtain the following:

7 $$k\text{'}=[\begin{array}{cc}\frac{3r}{3r+70\sqrt{2}} & 0\\ 0 & \frac{3r+70\sqrt{2}}{3r}\end{array}]k$$

**Figure 8:**
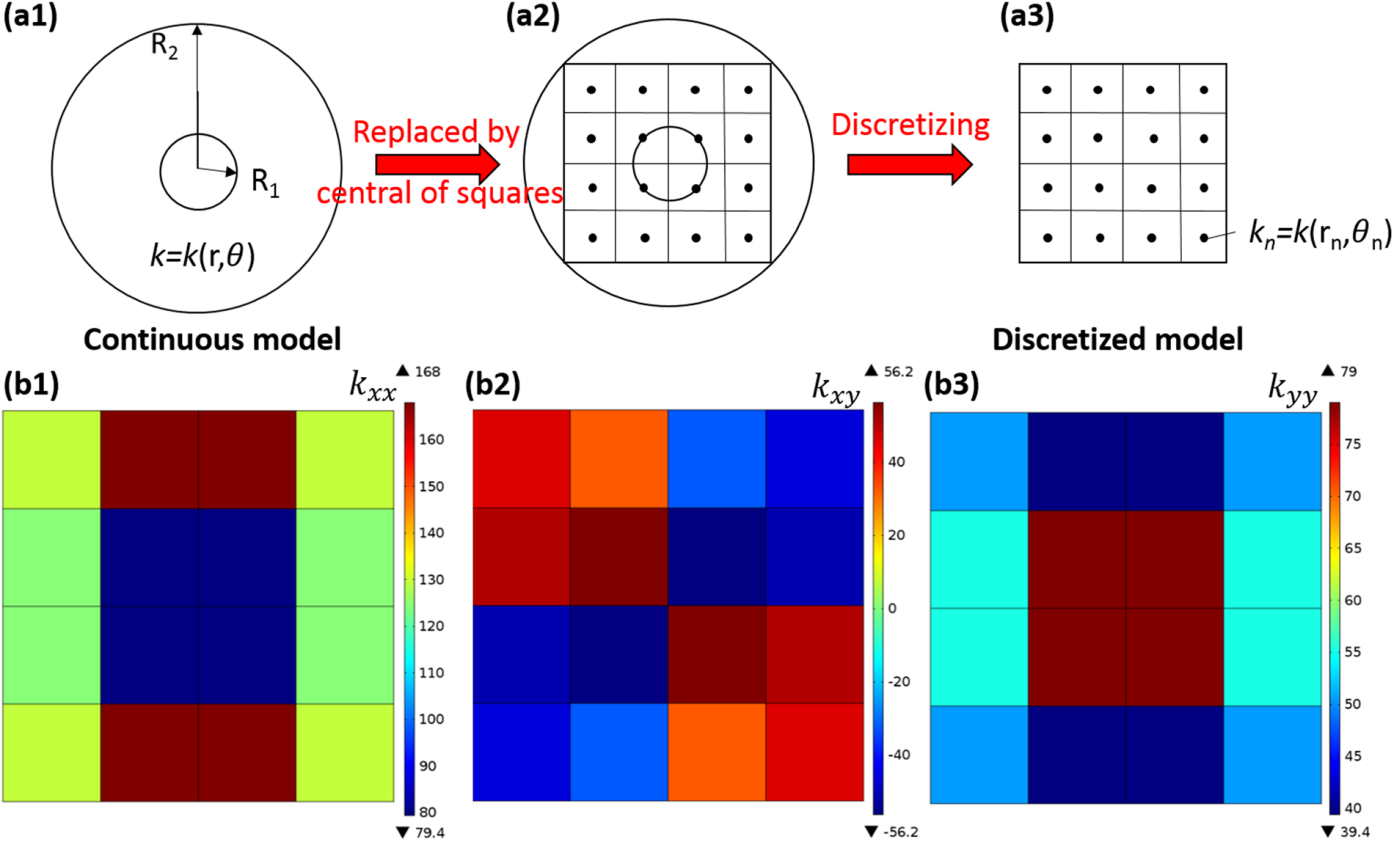
Design principle and anisotropic thermal conductivity of discretized thermal cloak. (**a1**) Continuous model (theoretical model). (**a2**) Selection of the central coordinate of each square to represent the anisotropic thermal conductivity of the whole model. (**a3**) Discretized model (effective model). (**b1**) *k*_*xx*_, (**b2**) *k*_*xy*_, and (**b3**) *k*_*yy*_.

We then discretize the thermal cloak into 4 × 4 unit-cell thermal shifters, as shown in Fig. 8(a1–a3). Subsequently, the equation (7) can be expressed in Cartesian coordinates as follows:

8 $${k^{\prime} }_{{\rm{C}}}=[\begin{array}{cc}(\frac{3r}{3r+70\sqrt{2}}){\cos }^{2}\theta +(\frac{3r+70\sqrt{2}}{3r}){\sin }^{2}\theta  & (\frac{3r+70\sqrt{2}}{3r}-\frac{3r}{3r+70\sqrt{2}})\cos \theta \sin \theta \\ (\frac{3r+70\sqrt{2}}{3r}-\frac{3r}{3r+70\sqrt{2}})\cos \theta \sin \theta  & (\frac{3r}{3r+70\sqrt{2}}){\sin }^{2}\theta +(\frac{3r+70\sqrt{2}}{3r}){\cos }^{2}\theta \end{array}]k$$

By substituting each center coordinate of the thermal shifters into the preceding equation, we obtain the desired thermal conductivity of each thermal shifter (See Supplementary Table S1).

Accordingly, we could obtain the required arrangement of each unit-cell thermal shifter by substituting the thermal conductivity in Supplementary Table S1 into the equation (5). The preceding task should be considered an optimization problem, which involves determining all the parameters in the equation (5) to obtain an optimum solution. In this instance, the unit cell thermal shifters in the thermal cloak are composed of layered, two-dimensional structures comprising copper as the high thermal conductivity and epoxy as the low conductivity material. Hence, we let *k*_1_ = 400*W*/*mK*, *k*_2_ = 0.3*W*/*mK*, d = 1 mm, and L = 35 mm in the equation (5) for manufacturing consideration, leaving only *L*_1_, *L*_2_, and θ to be determined. For the chosen parameter, the current optimization problem is subject to the following box constraints:

9 $$2\le {\varnothing }_{constraints1}={l}_{1}^{(u)}\le 7,\,{\rm{u}}=1,\ldots ,{\rm{N}}$$

10 $$2\le {\varnothing }_{constraints2}={l}_{2}^{(u)}\le 7,\,{\rm{u}}=1,\ldots ,{\rm{N}}$$

11 $$-\pi /2\le {\varnothing }_{constraints3}={\theta }^{(u)}\le \pi /2,\,{\rm{u}}=1,\ldots ,{\rm{N}}$$

By solving the preceding optimization problem, we can obtain the exact parameters described in the equations (9)–(11) (See Supplementary Table S2). As a consequence of the optimal parameters shown in Supplementary Table S2, the effective thermal conductivity varies inside the different unit-cell thermal shifters, as shown in Fig. 8(b1–b3), and is generally anisotropic.

To obtain the anisotropic thermal conductivity of the MMT-based TCC, we rotated the thermal conductivity in the equation (6) by 90°, identical to the angle rotated by the unit cell of rotating squares relative to each other (the tunable mechanism of the MMT-based TCC is displayed in Supplementary). Thus, we obtain the following:

12 $${k}_{\psi }=\frac{{J}_{\psi }{k}_{Theory}\cdot Trans({J}_{\psi })}{{\rm{\det }}({J}_{\psi })}$$

where

13 $${{\rm{J}}}_{{\rm{\psi }}}=[\begin{array}{cc}\cos ({\rm{\psi }}) & \sin ({\rm{\psi }})\\ -\sin ({\rm{\psi }}) & \cos ({\rm{\psi }})\end{array}],\,{\rm{\psi }}={0}^{0}\,{\rm{or}}\,\,{90}^{0}$$

In the equations (12) and (13), when the rotating angle, ψ, is 0°, *k*_*ψ*_ is the anisotropic thermal conductivity of the thermal cloak. However, when the rotating angle is 90°, *k*_*ψ*_ is the anisotropic thermal conductivity of the thermal concentrator. Hence, for other MMT-based thermal metamaterials with tunable functionalities, the anisotropic thermal conductivity can be obtained using the coordinate transformation technique, which involves rotating the original thermal conductivity by 90°.

### Data availability statement

The datasets generated during and/or analyzed during the current study are available from the corresponding author on reasonable request.

## Electronic supplementary material

Supplementary information

Video of MMT-based TCC
