# Supplementary material for: Realization of a thermal cloak–concentrator using a metamaterial transformer
Source: Sci Rep. 2018 Feb 6;8:2493. doi: 10.1038/s41598-018-20753-y (PMC5802869; doi:10.1038/s41598-018-20753-y)
Supplement: Supplementary file 1 — Supplementary information [file 41598_2018_20753_MOESM1_ESM.pdf]

Supplementary Information

Realization of a thermal cloak–concentrator using a metamaterial transformer

Ding-Peng Liu[1], Po-Jung Chen[1], Hsin-Haou Huang[1*]

[1]Department of Engineering Science and Ocean Engineering,
National Taiwan University
Taipei 106, Taiwan
*hsinhaouhuang@ntu.edu.tw

1. Evaluation of thermal resistance due to contact interface

To simulate the thermal resistance due to the contact interface, we created a 0.1mm thin layer in between adjacent thermal shifters in the simulation models, as shown in Figure S1. The thermal resistance highly depends on the property of the chosen thermal compound and the roughness of the copper interfaces. If ideal contact interface (smooth interface) is assumed, the thermal conductivity k is then set equal to $1\ \mathrm{W/(mK)}$ based on the product sheet provided by the manufacturer. Practically, the roughness of the interface is present, hence the thermal conductivity should become lower than that of the ideal case. In Figure S2, the temperature profiles resulted from the simulation and experiment are plot. In the figure, the simulation results assume various thermal conductivity values. The results show that the curve with k=0.5 $\mathrm{W/(mK)}$ is closest to that of the experimental data. The thermal resistance with rough contact interface is calculated as $57.1\ \mathrm{K/W}$ using Fourier's Law for heat conduction.

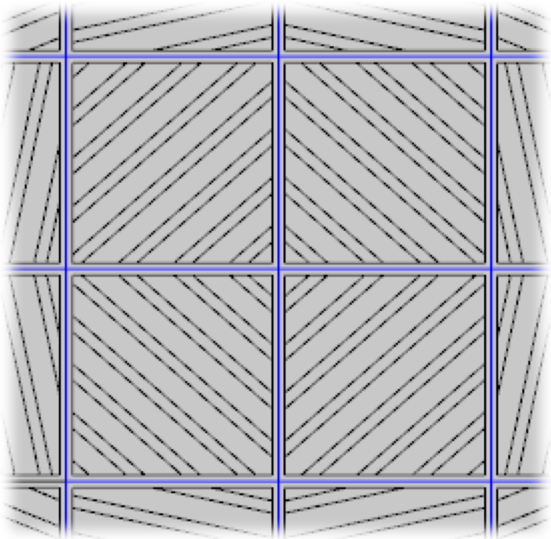

Figure S1. Thin layers in between the adjacent unit celss in the simulation model representing the contact interfaces.

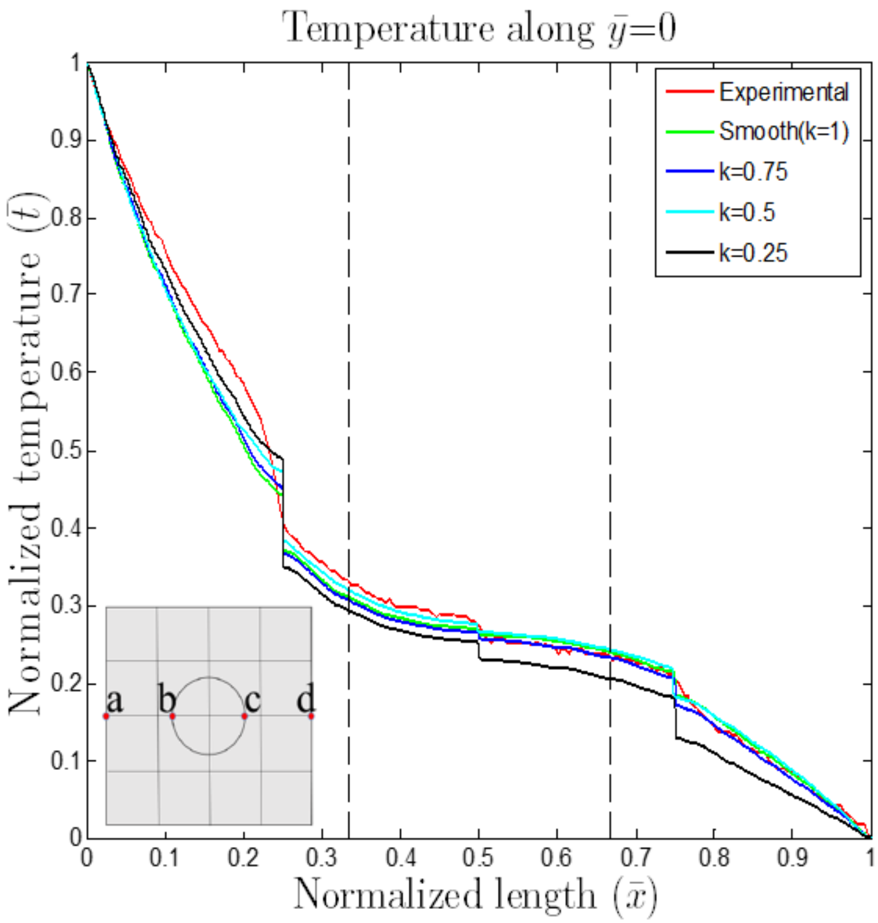

Figure S2. Temperature along $\bar{y} = 0$ of simulated and experimental results in 0° rotated TCCs. The simulated results are set with various thermal conductivity (k = 1, 0.75, 0.5, and 0.25).

2. Possible joint designs of the metamaterial transformer (MMT)

In the present experimental setup, we used plastic tapes to simplify the mechanical joints for the integrity of the MMT. Additionally, we proposed and illustrated options for the design of the mechanical joints in Figure S3. For the present design, the thermal shifters composed of copper and epoxy were placed on top of the wooden base connected with joints. The adjacent thermal shifters were connected and filled with thermal compound. The proposed options shown in the figure demonstrate that the joints can be designed with or without the bases.

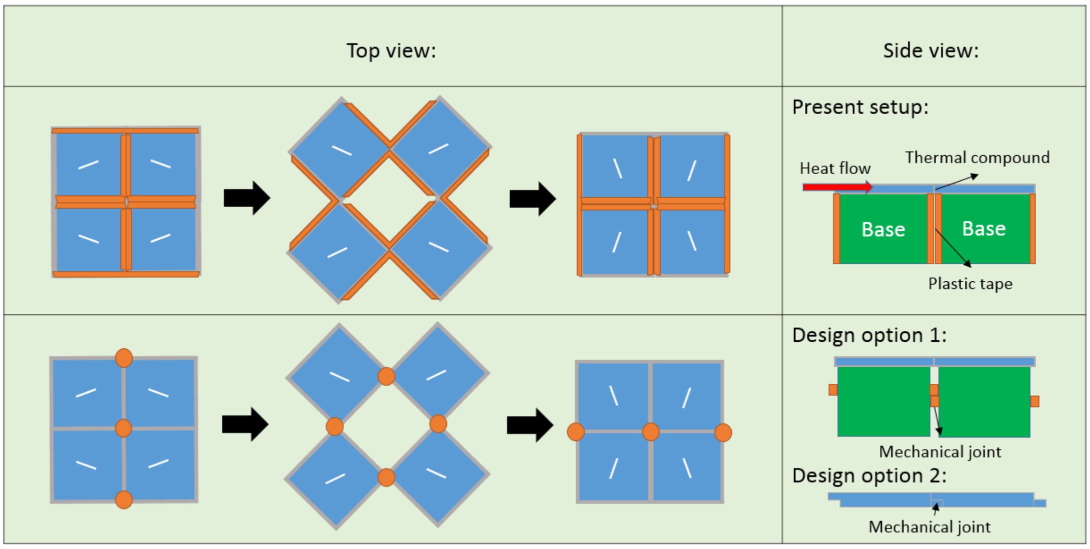

Figure S3. Schematic of possible joint designs of the metamaterial transformer (MMT).

3. Models of the unit-cell thermal shifter with contact interfaces

The top and the bottom boundaries were defined as the insulators, with the left boundary and the right boundary assigned the temperature 1K and 0K, respectively, as shown in Figure S4. In Figure S4(a), the theoretical model was assigned with the anisotropic thermal conductivity obtained from the equation (5), and Figure S4(b) presents the corresponding effective model with d= 1mm, $k_1 = 400W/(mK)$, $k_2 = 0.3W/(mK)$ and $L_a = L_b = 35$mm.

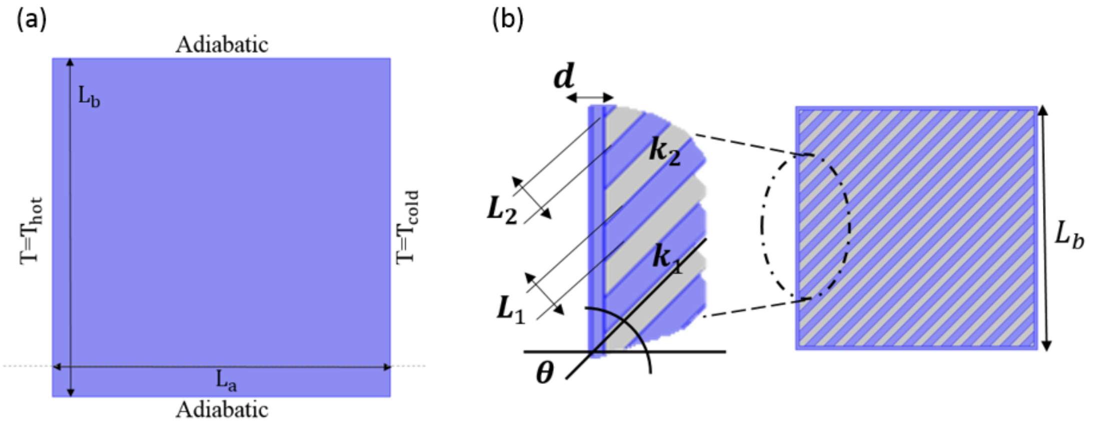

Figure S4. Illustration of a unit-cell thermal shifter: (a) The theoretical model and (b) The effective model.

First, we compared the results of the theoretical and effective models by letting L_2/L_1 = 1/3.5, 1, 3.5 and $\theta = 15°$. The simulated temperature profile and the isothermal lines are shown in Figure S5. From Figure S5(a) ~ (f), the pattern of the isothermal lines nearby the upper and lower boundaries of the thermal shifter when L_2/L_1 becomes greater indicates a rapid change in temperature.

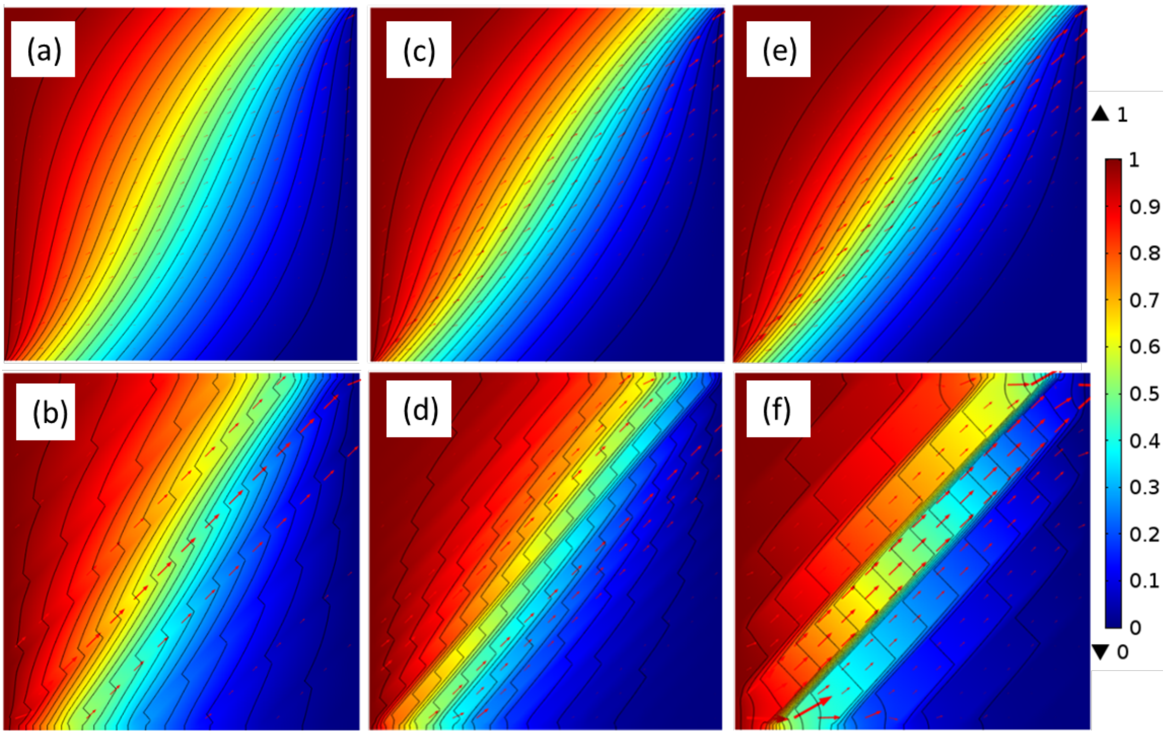

Figure S5. Temperature profile and isothermal lines of the unit-cell thermal shifters with various L_2/L_1 in the theoretical model: (a) L_2/L_1 = 1/3.5, (c) L_2/L_1 = 1, and (e) L_2/L_1 =3.5, and the effective model: (b) L_2/L_1 = 1/3.5, (d) L_2/L_1 = 1, and (f) L_2/L_1 =3.5.

We then compared the results of the theoretical and effective models by letting $\theta = 15^{\circ}, 30^{\circ}, 45^{\circ}$ and $l_1 = l_2 =$ 2mm. The simulated temperature profile and the isothermal lines are shown in Figure S6. From Figure S6(a) ~ (f), the heat flux (red arrows) are much inclined when θ becomes greater. The proposed anisotropic thermal conductivity of the unit-cell thermal shifter with contact interfaces was verified by comparing the temperature profile and isothermal lines of the corresponding effective model. Thus, we can design a discretized thermal cloak-concentrator (TCC) based on the proposed anisotropic thermal conductivity of the unit-cell thermal shifter with contact surfaces.

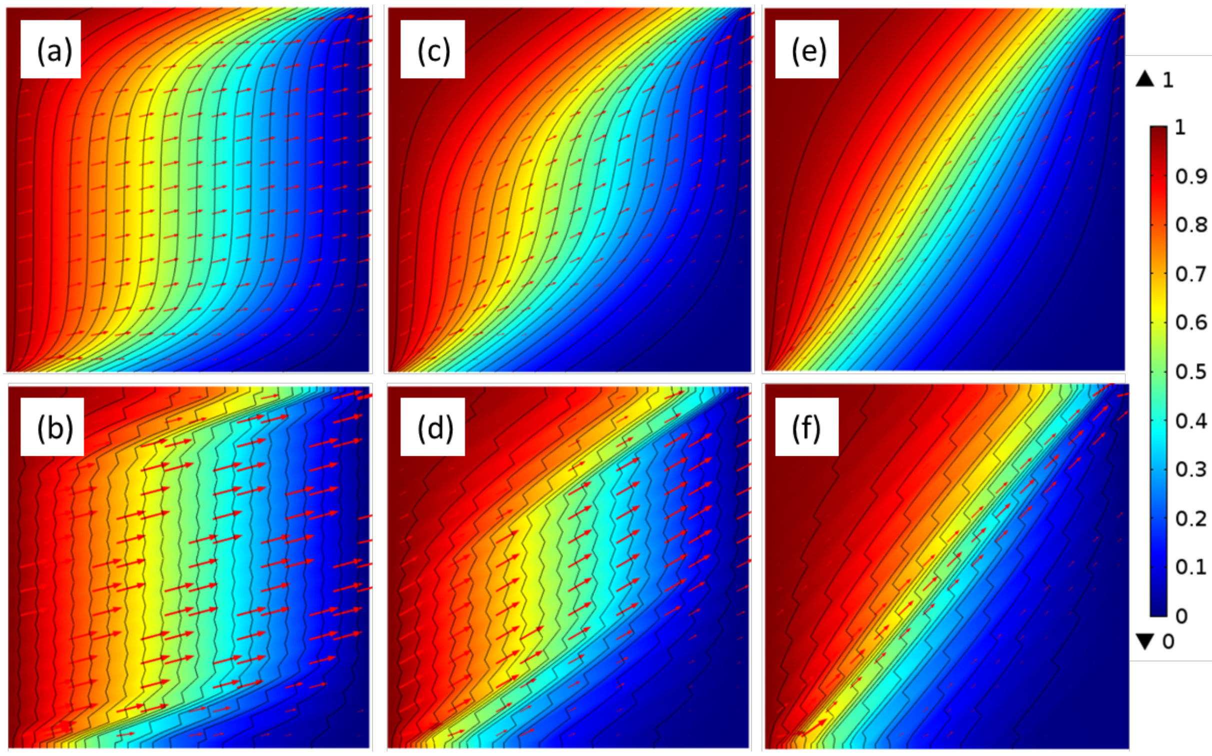

Figure S6. Temperature profile and isothermal lines of unit-cell thermal shifters with various θ in the theoretical model: (a) $\theta = 15^{\circ}$, (c) $\theta = 30^{\circ}$, and (e) $\theta = 45^{\circ}$, and the effective model: (b) $\theta = 15^{\circ}$, (d) $\theta = 30^{\circ}$, and (f) $\theta = 45^{\circ}$.

4. Table of the position and the desired thermal conductivity of each thermal shifter

By substituting each center coordinate of the thermal shifters into the equation (8), we obtain the desired thermal conductivity of each thermal shifter, as shown in Table S1. Accordingly, we could obtain the required arrangement of each unit-cell thermal shifter by substituting the thermal conductivity in Table S1 into the equation (5). The preceding task should be considered an optimization problem, which involves determining all the parameters in the the equation (5) to obtain an optimum solution.

(x,y)	k(x,y)	(x,y)	k(x,y)
(-52.5，-52.5)	$\begin{bmatrix} 1.07 & -0.40 \\ -0.40 & 1.07 \end{bmatrix} k$	(17.5，-52.5)	$\begin{bmatrix} 1.65 & 0.36 \\ 0.36 & 0.68 \end{bmatrix} k$
(-52.5，-17.5)	$\begin{bmatrix} 0.68 & -0.36 \\ -0.36 & 1.65 \end{bmatrix} k$	(17.5，-17.5)	$\begin{bmatrix} 20.51 & 20.48 \\ 20.48 & 20.51 \end{bmatrix} k$
(-52.5，17.5)	$\begin{bmatrix} 0.68 & 0.36 \\ 0.36 & 1.65 \end{bmatrix} k$	(17.5，17.5)	$\begin{bmatrix} 20.51 & -20.48 \\ -20.48 & 20.51 \end{bmatrix} k$
(-52.5，52.5)	$\begin{bmatrix} 1.07 & 0.40 \\ 0.40 & 1.07 \end{bmatrix} k$	(17.5，52.5)	$\begin{bmatrix} 1.65 & -0.36 \\ -0.36 & 0.68 \end{bmatrix} k$
(-17.5，-52.5)	$\begin{bmatrix} 1.65 & -0.36 \\ -0.36 & 0.68 \end{bmatrix} k$	(52.5，-52.5)	$\begin{bmatrix} 1.07 & 0.40 \\ 0.40 & 1.07 \end{bmatrix} k$

(-17.5，-17.5)	$\begin{bmatrix} 20.51 & -20.49 \\ -20.49 & 20.51 \end{bmatrix} k$	(52.5，-17.5)	$\begin{bmatrix} 0.68 & 0.36 \\ 0.36 & 1.65 \end{bmatrix} k$
(-17.5，17.5)	$\begin{bmatrix} 20.51 & 20.49 \\ 20.49 & 20.51 \end{bmatrix} k$	(52.5，17.5)	$\begin{bmatrix} 0.68 & -0.36 \\ -0.36 & 1.65 \end{bmatrix} k$
(-17.5，52.5)	$\begin{bmatrix} 1.65 & 0.36 \\ 0.36 & 0.68 \end{bmatrix} k$	(52.5，52.5)	$\begin{bmatrix} 1.07 & -0.40 \\ -0.40 & 1.07 \end{bmatrix} k$

Table S1. Table of the position and the desired thermal conductivity of each thermal shifter.

5. Table of global optima of the thermal shifters for the thermal cloak

Solving the preceding optimization problem results in the exact parameters described in the equations (9)~(11) (see Table S2). The corresponding number of the unit-cell thermal shifters are shown in Figure S7.

**No.**	1	2	3	4	5	6	7	8
**θ**	-22°	-12°	12°	22°	-12°	-41°	41°	12°
l_1**(mm)**	2							
l_2**(mm)**	4							
**No.**	9	10	11	12	13	14	15	16
**θ**	12°	41°	-41°	-12°	22°	12°	-12°	-22°
l_1**(mm)**	2							
l_2**(mm)**	4							

Table S2. Table of global optima of thermal shifters for the thermal cloak.

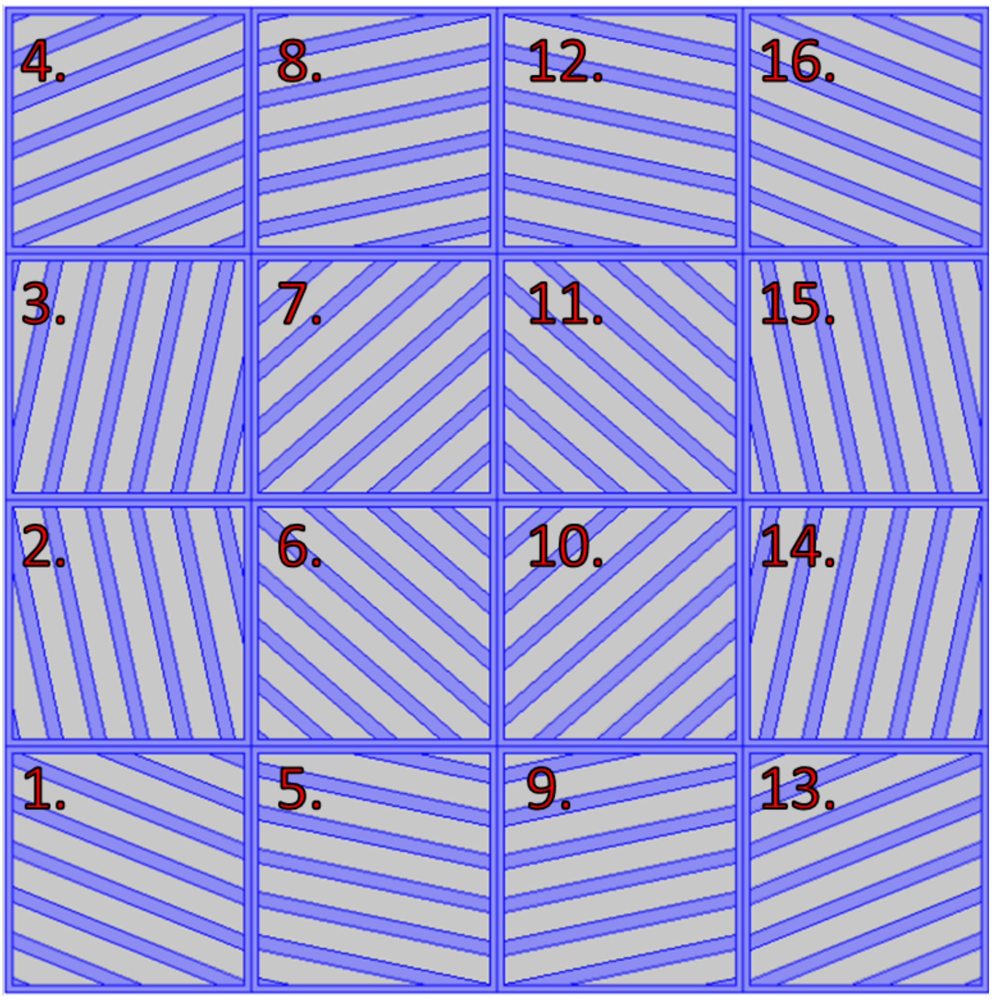

Figure S7. Schematic of the discretized thermal cloak composed of a 4 × 4 unit-cell thermal shifters.
